# Basic human values and safety in geriatric organizations: a study of state regulations in Israel

**DOI:** 10.1186/s13584-026-00791-y

**Published:** 2026-11-06

**Authors:** Keren Semyonov-Tal, Eldad Davidov

**Affiliations:** 1 Department of Social and Health Sciences, Bar-Ilan University, Ramat-Gan, Israel; 2 Institute of Sociology and Social Psychology, University of Cologne, Cologne, Germany

**Keywords:** Values​, Safety, Geriatric hospitals, Qualitative methods, Israel healthcare

## Abstract

**Background:**

The study explores how state regulations can encourage and influence organizational norms, such as safety in geriatric healthcare.

**Methods:**

Using thematic analysis, we focused on patient safety as an organizational goal by analyzing the content of 147 regulations related to geriatric treatment. The analysis was framed within Schwartz’s theory of basic human values.

**Results:**

Three main themes were identified: patient safety, staff safety, and safety and risk management. The findings show that safety-related values are systematically embedded in Israeli geriatric regulations and offer practical strategies for enhancing safety outcomes in geriat​ric hospitals.

**Conclusions:**

The study offers three main contributions. Firs​t, it applies an individual-level value theory to explain organi​zationa​l objectives in a health policy context. Second, it provides a t​hree-level regulatory framework (micro, meso, macro) that enables more targeted and specialized policy development. Third, it demonstrates that well-written regulations can serve as detailed operational tools that improve nonmedical aspects of care and advance organizational goals.

## Background

Values can be viewed as guiding principles illuminating paths that represent major aspirations and principles [1–7]. According to Schwartz [2], values contain six main features: (1) beliefs closely connected to emotions; (2) desirable goals that drive action; (3) principles that are not limited to specific actions or situations; (4) standards that guide the evaluation of actions, policies, people, and events; (5) relative prioritization, creating thus, a system of priorities; and (6) influence on individuals’ behavior and attitudes. In health settings, state regulations serve as an organizational compass, guiding staff conduct and distinguishing appropriate from inappropriate actions [8]. Written regulations can streamline processes, improve treatment, manage risks, reduce costs, and promote organizational goals, norms, and values [9–15]. The present study aims to improve understanding of how state regulations influenc​e organizational behavior by examining, for the first time, the role of geriatric regulations in Israel in promoting patient safety for elderly patients, framed within Schwartz’s theory of values.

Although existing international literature has examined the effects of regulatory stringency and staffing regulations on nursing home quality and resident safety outcomes [16–19], studies have not analyzed the content of geriatric care regulations through a theoretical values framework, nor has any comparable study been conducted in the Israeli context. The present study addresses this gap.

### The universal structure of basic human values

Schwartz [3–4] identified a series of core values that individuals recognize and prioritize. According to the theory, 10 abstract and specific values are universally identifiable across different cultures. These values can be grouped into four high-order value dimensions. The dimension of Openness to change includes the specific values of Stimulation and Self-direction. It opposes the higher-order value of Conservation, which includes the more specific values of Tradition, Conformity, and Security. Self-transcendence encompasses Universalism and Benevolence values and stands in contrast to Self-enhancement, which comprises Power and Achievement. Hedonism is a specific value that falls between Openness to change and Self-enhancement.

**Fig. 1:**
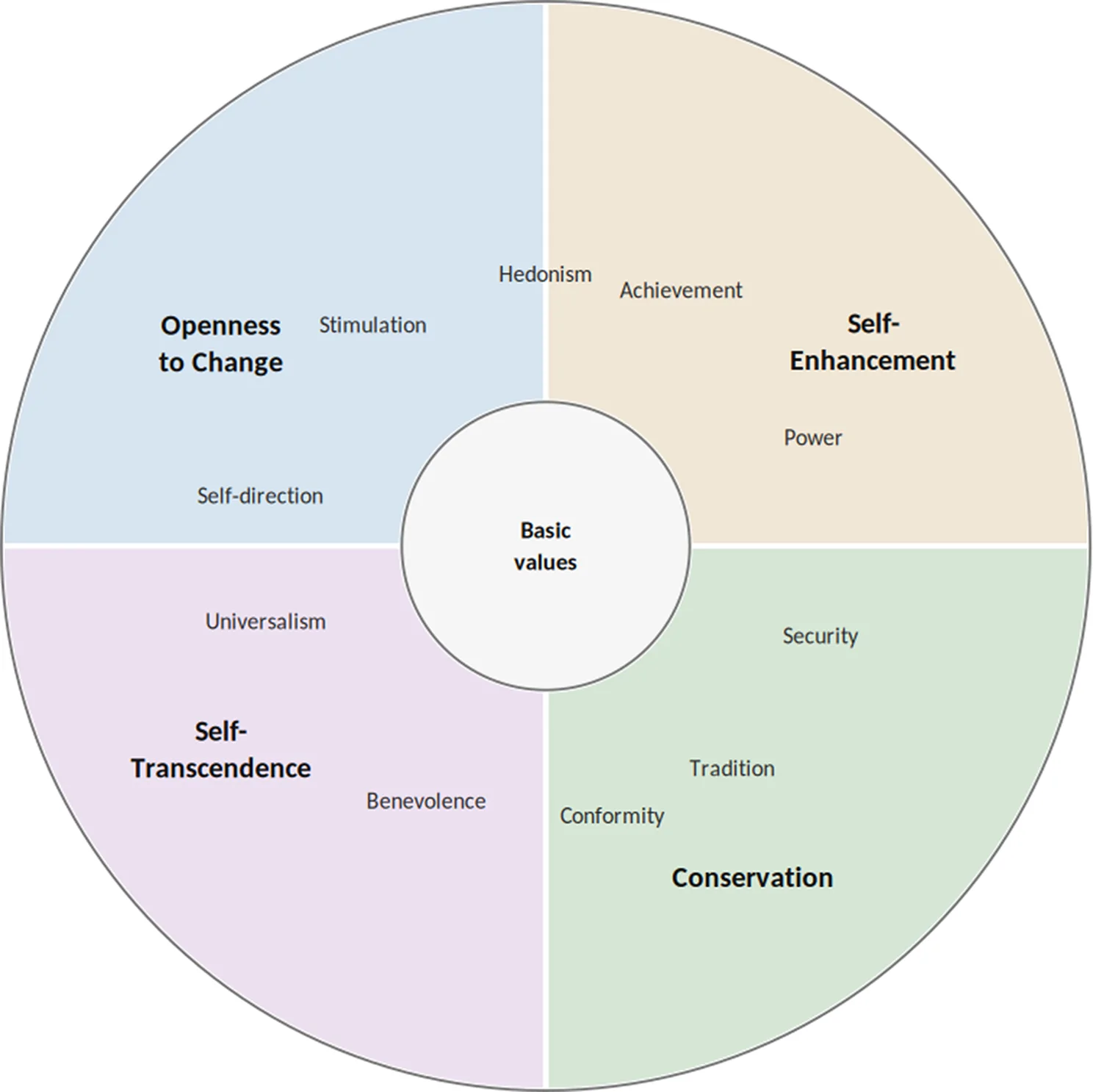
Schwartz’s values theory circular diagram

These four higher-order value dimensions form a continuum organized around a circle (see Fig. 1). Values closer to each other are more compatible, while opposing values contradict each other’s goals. According to the theory, individuals find it challenging to align their behavior with opposing values and are more likely to act according to the values they prioritize.

Schwartz’s theory was selected for several reasons. First, it has been empirically validated across more than 80 cultures, making it one of the most cross-culturally robust frameworks for studying values [3–7]. Second, the theory’s unique structure (i.e., encompassing 10 distinct values organized into four higher-order dimensions) provides a comprehensive lens through which to examine organizational goals such as safety. Third, unlike domain-specific value frameworks, Schwartz’s theory is designed to capture universal motivational goals, making it well-suited for analyzing regulatory documents aimed at diverse populations. It is worth noting that the term “basic human values” carries diverse meanings across disciplines (such as political, legal, and social). Yet, in this paper, the term is employed strictly within the Schwartz theoretical framework, referring to the motivationally distinct values he identified as universal across cultures [3–7].

**Table 1:** Schwartz’s theory of values (1992, 1994) [3–4]

Higher-order dimensions	Original 10 values	Definition
Openness to change	Self-direction	Independent thought and action: choosing, creating, and exploring
	Stimulation	Excitement, novelty, and challenge in life
	Hedonism	Pleasure and gratification for oneself
Self-enhancement	Achievement	Personal success through demonstrating competence according to social standards
	Power	Social status and prestige, control, or dominance over people and resources
Conservation	Security	Safety, harmony, and stability of society, relationships, and self
	Tradition	Respect, commitment, and acceptance of the customs and ideas provided by traditional culture
	Conformity	Restraint of actions, inclinations, and impulses that may upset or harm others
Self-transcendence	Universalism	Understanding, appreciation, tolerance, and protection for the welfare of all people and nature
	Benevolence	Preserving and enhancing the welfare of those with whom one is in frequent personal contact

The theory’s critical points primarily focus on individual values, but it also offers insights into how personal values shape social dynamics and group behavior [6, 7]. Even though Schwartz’s theory does not directly deal with organizational values, we contend that the implications of individual values can be extended to organizational settings. In the following, we focus on safety in health organizations as part of the security value, which belongs to the higher-order value dimension of conservation (see Table 1).

### Safety

According to the World Health Organization (WHO), safety in health settings is a major mechanism for preventing harm to patients and reducing risks of physical or mental maltreatment [20]. This includes medication safety, infection control, surgical safety, and system safeguards to ensure patients’ security [21, 22]. The goal of prioritizing hospital safety is to enhance the quality of care and minimize harmful events or errors [23]. This is crucial for maintaining and improving trust and effectiveness [20, 21, 24]. According to the WHO Patient Safety Curriculum Guide [23], safety topics should be integrated into existing medical curricula. In a comprehensive review paper, Ji and colleagues [25] present a systematic analysis of 57 papers on safety in healthcare settings. The paper highlights the role of education in fostering safety in healthcare and calls for optimizing educational strategies for medical staff. Likewise, interprofessional teamwork is seen as a significant issue in care and can improve patient safety [26].

Additional safety problems in healthcare settings include medication errors, surgical errors, and medical errors such as misdiagnosis, incorrect treatment, and failure to provide care, which can harm patients [27]. Infections are a significant problem, often resulting from inadequate infection control practices. Patient identification errors, such as wrong-site surgeries and incorrect medication administration, are recognized as serious safety issues [20, 21]. Poor communication between health staff and patients can increase misunderstandings and errors in care [26].

Previous studies examining the role of staff safety in health settings found that the well-being of healthcare staff is strongly associated with it. Baker and colleagues [28] observed that staff members protect themselves and others by following several key guidelines. This includes using protective equipment such as long-sleeved clothing, masks, and gloves to minimize exposure to infections. Hygiene practices, such as washing hands thoroughly with soap or frequently using an alcohol-based hand sanitizer, are also vital. Pre- and post-shift showers or changing clothes before and after shifts is advised to lower the risk of carrying viruses home. Regular cleaning and disinfecting of personal items and workspaces are also crucial to minimize the risk of virus transmission. Adhering to these guidelines can significantly reduce the risk of infection for healthcare staff [28]. Studies also found that medical staff burnout will likely harm staff and patients’ safety and well-being [29–31]. Additionally, issues in healthcare systems and processes, such as inadequate staffing, insufficient protocols, and insufficient training, can contribute to unsafe practices [20–23].

Beyond the WHO, patient safety in medical care has been addressed by a range of professional and regulatory bodies. The American Medical Association (AMA) has long championed patient safety initiatives, emphasizing the physician’s ethical responsibility to minimize harm [32]. Other influential bodies include the Joint Commission International (JCI), which accredits healthcare organizations worldwide based on safety and quality standards [33], and the Institute for Healthcare Improvement (IHI), which has developed widely adopted frameworks for reducing medical errors and improving care systems [34].

Safety is also intrinsically linked to risk management in healthcare organizations. Effective risk management reduces the frequency of adverse events and, consequently, the incidence of medical malpractice claims and associated litigation costs [35]. In Israel, organizations such as Madanes Insurance Agency, Inbal Insurance Company, and the Quality and Safety System at the Ministry of Health (MOH) focus on safety and risk management in healthcare [36].

Geriatric patients face distinct safety challenges that set them apart from the general hospital population. Falls represent one of the most serious and prevalent risks among older adults in institutional settings, often resulting in fractures, prolonged hospitalization, and increased mortality [37–39]. Elderly patients are also disproportionately vulnerable to healthcare-associated infections, due to age-related immune decline and the frequent use of invasive procedures [40]. Medication safety presents a particular challenge in geriatric care, as older patients often are subject to an increased risk of adverse drug interactions and dosing errors [41]. These population-specific safety challenges underscore the importance of examining geriatric-specific regulations, as general safety frameworks may be insufficient to address the needs of this vulnerable group.

### The Israeli healthcare system and geriatric care

Israel has a comprehensive network of nationwide clinics, public hospitals, and preventive medical services [42]. The government primarily funds the healthcare system and provides national health insurance to all permanent residents, regardless of gender, ethnicity, or religion [43]. The Patient’s Rights Act of 1996 [44] guarantees that all residents receive respectful and dignified medical care and safeguards patients’ legal rights [8, 45].

Israeli Geriatric hospitals are governed by the 1940 People’s Health Ordinance [46] and the 1966 People’s Health Regulations [Registration of Hospital] [47].^1^ Any medical facility, including geriatric hospitals, must obtain approval from the Department of Health before establishing or starting a medical operation. As per these laws [46, 47], a geriatric hospital is a medical institution designed to provide specialized medical care and support for individuals aged 65 and over. Geriatric hospitalization includes long-term care, such as nursing and active geriatric care, complex nursing, geriatric rehabilitation, and hospice care. It is important to note that alongside standalone geriatric hospitals, geriatric wards also exist within general hospitals, providing specialized care for older adults within a broader acute-care setting.

In recent years, Israel has experienced a significant demographic change with a rapidly growing elderly population, similar to other developed nations. This shift presents challenges and opportunities for adapting geriatric health policies to meet the needs of older adults [48]. The regulatory health environment in Israel is distinct from that of other developed countries. The Ministry of Health serves multiple roles as a regulatory authority, international trade negotiator, and supporter of the pharmaceutical and health industries, creating a complex partnership that balances regulatory functions with support for health-related goals [49]. This unique blend of public health initiatives and industry support is rarely observed in other developed countries [49]. Furthermore, while the Ministry of Health functions as the regulatory authority overseeing healthcare standards and practices in Israel, it also plays an essential role as a healthcare provider [50–51].

In Israel, some policies have adapted to the needs of older adults, but others remain inadequate. For instance, Israeli legislation offers limited support for family caregivers, indicating a need for more comprehensive geriatric care policies to support informal care systems alongside formal care [52]. Unlike other countries, the Ministry addresses elder abuse through legal and social frameworks, highlighting the need for culturally sensitive approaches [53]. The introduction of specialized roles for nurse practitioners in geriatrics reflects Israel’s commitment to comprehensive geriatric care [54]. Similar to practices in countries such as the USA and Australia, Israeli strategies for older adult fall prevention have shifted toward international standards [37].

Safety issues in Israel’s geriatric institutions primarily focus on falls, inadequate staffing, and managing complex health conditions. Falls are a significant concern, often resulting in severe injuries for elderly patients and increased healthcare costs [38, 39]. Insufficient staffing levels and inadequate training further contribute to the poor quality of care and safety measures for geriatric patients [40]. Staff in Israeli geriatric institutions face challenges in managing resident behavior, frequently resorting to physical restraints and psychotropic medications, which can worsen safety issues such as falls, pressure sores, and adverse drug events [55]. Furthermore, frail individuals are at a higher risk for infections, thus requiring proactive management strategies [40]. The Ministry of Health regularly inspects geriatric facilities to address violations such as safety violations, yet future research of the audit reports is needed to identify the types of violations.^2^

## Methods

In 2007, the Ministry of Health published an updated version of procedures for geriatric hospitals.^3^ The procedures, publicly available data^4^. It include 147 formalized regulations for medical personnel. The documents, numbering over 1,000 pages, contain detailed quality control, safety, and provider-patient relationship regulations. For the present research, we analyzed 147 procedures and circulars from 2007 to the present day to examine whether, how, and to what extent regulations related to geriatric treatment in Israel prioritize patient safety. While safety considerations in geriatric institutions existed prior to this period, most regulations were updated between 2007 and the present.

To address the research questions, we adopted a qualitative content analysis approach [56, 57] to analyze the written information. The analysis involved manual and software-based AI methods (atlasti.com) to identify recurring codes and connections. The coding process involved the extraction of both literal and interpretative codes. The data obtained from the coding process were structured into potential subthemes that were then discussed with colleagues to ensure the themes and subthemes formed a consistent and logical arrangement. Relevant quotations were translated from Hebrew to English for the purpose of the discussion here. The final findings were documented using themes, relevant notes, and data excerpts to ensure credibility and transferability. We conducted the coding process iteratively. Initially, we analyzed the data using classic thematic analysis without considering the specific literature on patient safety. However, as we progressed with the coding, three main themes emerged, which were subsequently examined and refined based on the interaction between the literature and research terrain. Codes that did not correspond to the defined themes of patient safety were excluded.

## Results

According to the Ministry of Health in Israel, safety in geriatric settings is one of the major and highly significant organizational goals [58]:

*Protecting the lives of patients and their safety in the hospital is one of the most important tasks that requires preventive actions and appropriate assessments (Procedure 3.1.4: Safety and emergency time)*

Table 2 li​sts safety themes that recurred throughout the data. Although patient safety is the dominant theme (3.1), two additional themes surfaced – staff safety (3.2) and risk management (3.3).

**Table 2:** Themes and subthemes of safety in geriatric hospitals

Theme	Subtheme	Description
Patient Safety	Patient well-being maintenance	Preventing errors and adverse events and maintaining patients’ physical and emotional well-being
	Fall prevention	Preventing falls by identifying and removing environmental hazards
	Infection prevention	Maintaining infection control practices to reduce pathogen transmission
	Medication error prevention	Ensuring proper medication management, storage, and administration
	Medical error prevention	Implementing practices to prevent adverse clinical outcomes and medical errors
	Patient misidentification prevention	Preventing mistakes or inaccuracies in patient identification
	Safety standard maintenance	Upholding and monitoring established safety standards
	Physical harm prevention	Implementing physical, mental, and financial safety measures
Staff Safety	Staff well-being maintenance	Addressing burnout and stress management to support staff health
	Occupational hazard prevention	Addressing occupational health risks and preventing physical injury to staff
Safety and Risk Management	Risk assessment and identification	Identifying and documenting personal and environmental risk factors
	Post-incident harm prevention	Discussing incidents and developing corrective actions to prevent future occurrences
	Continuous improvement	Implementing continuous improvements in safety practices

### Patient safety

Recurrent subthemes uncovered in our analysis include patient well-being, fall prevention, infection prevention measures, medication errors, medical errors, patient identification errors, safety standards, and physical safety.

According to state regulations, ensuring the well-being of patients is a key organizational goal:

*In order to improve the quality of care*,* the nurse in a geriatric hospital will ensure that the transfer of a patient to the hospital is done professionally while taking care of his/her safety and well-being from the time s/he leaves the geriatric hospital until his/her hospitalization in a general hospital and his/her return to the geriatric hospital. (Procedure 2.2.3: Referral of a patient to a general hospital)*

Creating a safe environment in geriatric hospitals involves a range of measures. The regulations emphasize maintaining a safe and secure environment where physical safety is prioritized:

*A safe and accessible environment promotes the patient’s functional abilities. (Procedure 0.4.8: Safety environment for the patient and prevention of accidents and falls)*

Ensuring the well-being of patients involves measures to prevent patients from harming themselves or others:

*Sometimes physical restraint of a patient is required as part of treatment and to prevent harm to the patient and his/her environment. (Procedure 0.4.8: Safety environment for the patient and prevention of accidents and falls)*

The responsibility to maintain the patient’s safety applies to all medical staff, family members, and visitors:

*The attendant is responsible for the patient’s safety and fulfilling his/her basic needs. (Procedure 2.2.3: Referral of a patient to a general hospital)*

Families play an essential role in improving patient safety:

*A patient’s family will be a partner and give their consent to the treatment plan prescribed for the patient to prevent repeated events or to increase the patient’s safety. (Procedure 2.3.5: Preventing falls and accidents among patients)*

Falls and accidents among the elderly are complex problems faced by health services worldwide [20, 21]. Therefore, the procedures provide detailed approaches to preventing falls and accidents among patients. These include taking steps to prevent hazards like slippery floors and ensuring sufficient lighting in hallways and common areas:

*The geriatric hospital will take the necessary actions and measures to ensure a safe environment and remove obstacles and environmental hazards that endanger the elderly. (Procedure 2.3.5: Preventing falls and accidents among patients)*

Regulations require developing specific protocols for high-risk activities such as fall prevention to ensure the well-being of elderly patients:

*The hospital administration will ensure the existence and proper maintenance of safety rails and handrails in the corridors*,* in the patient services*,* and everywhere as required in the building program. (Procedure 3.1.4: Safety during an emergency)*

These protocols help minimize the risk of accidents:

*A safe and accessible environment* … *will minimize the risks of accidents and falls which have a decisive effect on the patient’s condition.* (Procedure 0.4.8: Safety environment for the patient and prevention of accidents and falls)

Maintaining cleanliness throughout the facility to prevent the spread of infections is emphasized in many of the documents:

*Each* *caregiver will observe hand hygiene before and after direct contact with the patient or his/her food and medicines.* *(Procedure 0.4.6: Prevention of infections)*

The hygiene of a patient’s environment includes disinfecting medical equipment, general cleaning and disinfection to reduce infections. Family and visitors are also required to adhere to the rules:

*A visitor to a patient staying in isolation is obliged to observe the same rule of hygiene as medical personnel and caregivers, including the use of gloves and a gown*. *(Procedure 0.4.6: Prevention of infections)*

Healthcare facilities employ various measures to minimize patient identification errors:

*The caregiver’s responsibility is to make sure that the following conditions are met: identification of the patient or the applicant by name, by at least two means (for example, name and ID number)* *(Circular 12/2017: Providing medical advice in a meeting without the presence of the patient in situations of acute illness)*

Proper medication man​agement, storage, and admini​stration are crucial to preventing medication errors:

*The proper storage of drugs in the wards and checking their storage is designed to improve the safety of drug therapy. (Procedure 8.0.3: Guidelines for storing medicines in geriatric hospital wards)*

Medical errors, such as incorrect treatment, are also​ significant. Given the complex medical needs of geriatric patients, healthcare providers must be vigilant and proactive in preventing and addressing medical errors to ensure the safety and well-being of older adults in their care:

*The training activities and the updating ​of professional knowledge will be carried out by the nursing director or a clinical supervisor*… *and will include … sharpening the skills of the entire nursing staff to prevent medical errors and promote the safety of treatment*. *(Procedure 2.1.3: Updating professional knowledge of the nursing staff)*

Safety protocols in geriatric hospitals involve raising awareness of safety measures:

*The goals of the procedure are to raise awareness of maintaining a safe environment and preventing falls/accidents and to guide hospital employees in the steps required to ensure a safe environment*,* each in their occupation and professional responsibility. (Procedure 0.4.8: Safe environment for the patient and preventing accidents and falls)*

The regulations emphasize safety standards, such as fire and electrical safety:

*Each establishment must have a valid fire extinguishing certificate* … ​*the maintenance person will ensure the integrity of the electrical equipment as well as the exposed electrical points*. *(Procedure 3.1.4: Safety during an emergency)*

The importance of safeguarding elderly patients from any form of abuse or violence, whether physical, emotional, or financial, is also highlighted. Violence and abuse are defined as:

*​Harm to the patient’s physical or mental well-being by using force or by hurting his/her feelings and causing mental suffering or by neglecting the patient. ​As well as financial abuse by using a patient’s money or property without permission. (Procedure 0.2.1: Definition of concepts)*

An example of this, Circular No. 20/12, extensively addresses the handling of acts of violence against geriatric patients while focusing on their vulnerability:

*When there is a suspicion of harm (violence*,* sexual harm) to a person hospitalized in one of these departments (i.e.*,* psychiatric or geriatric ward)*,* the suspicion of harm must be reported to the police or the welfare officer*,* as well as a notice of an unusual event must be issued to the Ministry of Health. (Circular No. 20/12: Treatment of suspected violence against the helpless treated in the health system)*

The regulations emphasize the need to protect elderly patients from all forms of abuse. This involves implementing measures to identify, address, and prevent any mistreatment or neglect of geriatric patients within healthcare settings:

*Any abnormality and/or fear of abuse of the elderly will be reported immediately. (Procedure 0.1.2: Patient rights)*

Patient safety also involves safeguarding against financial abuse. For instance, Circular 63/2002 outlines specific procedures for managing patient deposit funds in psychiatric and geriatric settings. As patients may be vulnerable, medical staff must safeguard their money and prevent misuse.

### Staff Safety

The data analysis underscored several recurring subthemes that emerged. These include staff well-being, physical safety, and occupational health.

Staff well-being in the context of safety in geriatric hospitals encompasses the hospital staff’s physical, mental, and emotional health. Reducing staff burnout is a strategy to promote staff-well-being:

*The social worker will be a partner in planning and carrying out events for the team to promote consolidation*,* empowerment*,* and prevention of burnout” (Procedure 4.0.1: Scope of duties of a social worker in the hospital and definition of his duties)*

The regulations emphasize maintaining a safe and supportive work environment, which reduces burnout, by providing adequate resources and training:

*The nurse in charge… will develop tools to identify and prevent burnout among the nursing staff who care for the elderly and nursing patients and will work to locate and reduce the causes of burnout and the cultivation of the human resource by providing constant support and training. (Procedure 2.1.3: Updating professional knowledge of the nursing staff)*

Inadequate infection control practices often lead to healthcare-associated infections, posing a significant challenge. Healthcare workers risk infection due to their exposure to a variety of serious infectious diseases. They may also be a source of transmission of diseases to patients. Therefore, the guidelines encourage the health system employees to receive vaccinations against serious diseases, thus protecting the patients and themselves against infection:

*The vaccination of the staff will preserve the health of the employees*,* will reduce absences due to illness and possible claims of the employees for work accidents due to the infection of the empl​oyees. (Procedure 0.4.11: Staff health and staff vaccinations)*

Prevention of staff infections and addressing occupational health risks, such as exposure to hazardous substances or infections, applies to all healthcare personnel:

*The health of the staff and vaccinations for the staff* … *apply to the hospital management and to all employees who may come into contact with patients or their body fluids. (Procedure 0.4.6: Prevention of infections)*

### Safety and risk management

Further analysis underscored the importance of risk assessment and identification, post-incident analysis, continuous improvement, incident reporting, and documentation.

​In geriatric hospitals, risk assessment can be utilized to identify and document personal and environmental risk factors such as falls, medication errors, and other incidents.

*The importance and uses of the medical record - a basis for risk management of patient safety. (Procedure 2.3.1: Nursing registration*,* documentation and reporting)*

According to the analysis, post-incident reviews, including discussing incidents and developing corrective actions, are recommended to prevent future issues and incidents:

*After repeated falls or accidents*,* a discussion will be held with the participation of the multi-professional team […]and propose corrective actions to prevent similar incidents from occurring in the future. (Procedure 2.3.5: Preventing falls and accidents among patients)*

Additionally, the documents emphasize the importance of striving for continuous improvement:

*An internal procedure guiding a safe environment will be prepared and implemented in the geriatric hospital. (Procedure 3.1.4: Safety in an emergency)*

## Discussion

In this study, we considered whether and how the goal of patient safety is rooted in medical regulations and how values defined with the framework of a theory are identified and prioritized by policymakers and ultimately articulated in governmental regulations as organizational directives​. The findings reported here support the premise that Schwartz’s theory of values [6, 7] offers a useful framework not only for understanding individuals’ values but also for understanding organizational values. Indeed, values at the individual level have clear implications for understanding the emergence of values at the organizational level and in group contexts [2]. The analysis also indicates that Israeli governmental regulations can be categorized along three levels: patient care (micro), healthcare institutions (meso), and healthcare policy (macro) [59].

### Patient safety

Patient safety is of the utmost importance in Israeli geriatric hospitals. It reflects the micro level of organizational behavior. This means that creating practices and procedures within the hospital at an individual level considerably influences patient well-being. Patient safety revolves strongly around the provision of security and secure conditions. However, safety is also associated with the goal of preventing errors. As such, it is directly connected to the value of Universalism [6, 7], particularly in terms of concern for people’s well-being. In the context of geriatric treatment, Universalism provides equitable care to all patients. Hence, all patients, regardless of their socioeconomic status, ethnicity, age, gender, or other considerations, should receive the same level of safety in their care. By adhering to safety regulations such as medication management or infection control, healthcare providers can uphold the principles of universalism, ultimately enhancing patient safety.

### Staff safety

The theme of staff safety in geriatric hospitals pertains to organizational behavior, a theme crucial for ensuring a safe and healthy work environment for staff members. Staff safety closely aligns with the values of Benevolence and Security [2, 3]. By prioritizing staff safety, hospitals demonstrate a commitment to their employees’ welfare. Creating a safe work environment aligns with the value of Security, which includes personal safety. Hence, Security is once again a key component that promotes a positive work environment and, eventually, patient care. When addressing the subthemes of staff safety, it is fundamental to consider the emotional toll due to the nature of geriatric patient care. Providing emotional support through counseling services, stress management programs, and peer support groups can help moderate burnout and promote staff well-being [31]. Similarly, fostering a supportive, safe workplace culture can enhance job satisfaction and reduce stress [30]. Addressing occupational health risks, such as virus exposure, is also vital to the value of Security.

### Safety and risk management

Safety and risk management in geriatric hospitals reflects organizational behavior at a meso level. By prioritizing safety and risk management, geriatric hospitals create a secure patient environment, improve the quality of care, and increase organizational efficiency. Safety and risk management can be linked to the values of Conformity and Tradition [6, 7]. Conformity encompasses adhering to rules and regulations that ensure safety, while tradition highlights respect for established practices that contribute to a stable environment. Risk assessment and continuous improvement in health organizations also relate to the Self-direction value [6, 7], particularly in independent thought and action. When empowered to think independently, health professionals can proactively identify risks and areas for improvement, enhancing patient safety and care quality. This sense of Self-direction fosters accountability, encouraging staff to engage in ongoing learning and innovative problem-solving. It also promotes collaboration, as input from diverse players enriches continuous improvement efforts [60]. Notably, aside from self-direction, all the values identified in the analysis belong to the theory’s higher-order social values of self-transcendence and conservation [6, 7]. This gap is significant, as empirical evidence shows that professional autonomy directly predicts safety-oriented behavior, including incident reporting, policy development, and quality improvement [60].

### Categorization of regulations

Categorizing governmental regulations into three levels in the context of geriatric healthcare provides a structured framework for identifying distinct areas of focus within a regulatory health system. The analysis presented in this study focuses on patient and staff safety at the micro and meso levels, while, at the macro level, it refers to broader health policies for geriatric healthcare. Dividing regulations into three levels enables more targeted and specialized policies on one hand while also helping to identify gaps or overlaps in the regulatory framework on the other hand. Hence, by doing so, policymakers can identify areas of the regulations that are overlooked or less emphasized. For instance, the Israeli geriatric regulations take a worker-focused approach to safety, which risks oversimplifying the complex realities present in the workplace environment. Thus, policymakers may want to highlight the dynamics of peer interactions in regulations, as they can significantly influence workplace safety behaviors. Other examples that receive less attention in the regulations and should garner policymakers’ focus include how effective leadership can reinforce safety practices and the significance of education and ongoing training related to safety issues in geriatric care settings [61].

While the analysis and Schwartz’s theory successfully demonstrate a valid connection between safety regulations and norms, they overlook the complexity of balancing safety with other essential values. For example, the framework presented emphasizes that the safety of patients and healthcare workers is fundamental for the well-being of older adults, many of whom may reside in geriatric hospitals for extended durations. However, these institutions are not only medical facilities demanding strict regulatory compliance but also the homes of residents who often remain there until the end of their lives. This duality amplifies the need for a careful balance between safety regulations and the flexibility to ensure residents’ high ​quality of life [62]. Another example not addressed in the regulations is the conflict between patient autonomy and safety. On one hand, physical restraints are used for patient safety; however, restraints compromise the patient’s well-being and autonomy [63]. Balancing patient consent and patient safety in geriatric care presents another complex challenge. On the one hand, respecting patient consent is vital for ensuring that elderly individuals retain their autonomy and have a say in their care decisions. On the other hand, safety is a critical consideration in geriatric care, and health organizations must confront the uncomfortable reality that many of these individuals may be faced with cognitive impairments or health issues that cloud their judgment [63].

## Conclusion, policy implications, and future recommendations

The study emphasizes the connection between patient safety and organizational values in healthcare systems, especially in geriatric care. It makes three significant contributions. First, it employs a theoretical model focused on individual values to clarify organizational objectives. Providing policymakers with conceptual tools, such as value-based frameworks, to better align regulations with organizational goals enables them to formulate regulations that improve the quality of care, resulting in better outcomes for elderly patients. Second, the study categorizes governmental regulations into three levels, micro, macro, and meso, creating an organized framework for pinpointing various focus safety areas (or lack thereof) within a regulatory health system. This framework can assist policymakers in creating more targeted and specialized policies while also addressing areas that are less emphasized or overlooked, as well as tackling dilemmas of competing values. Third, the findings indicate that well-written regulations can provide detailed operational instructions that improve the nonmedical aspects of healthcare and help achieve organizational objectives. However, the findings reported here do not clarify whether and to what extent safety guidelines are sufficiently implemented in geriatric institutions. Future studies should examine how these values are practically applied in hospital settings in Israel and other societies.

## Data Availability

The data supporting this study’s findings are available on request from the corresponding author.

## Footnotes

1 The People’s Health Ordinance of 1940, established by the British Mandate, regulates the healthcare system, particularly health institutions like hospitals for the elderly. It requires medical institutions to obtain authorization from the Ministry of Health for establishment, conversion, expansion, or bed additions. The ordinance has been amended over the years, notably in 1991 and 2003, to enhance the quality and oversight of public health services.

2 https://www.gov.il/he/pages/geriatric-institutions-reports.

3 The procedures file consists of nine chapters. The first chapter provides an overview of geriatric treatment. Subsequent chapters include professional fields (i.e., medicine, nursing, administration, social work, nutrition, physiotherapy, communication clinics, occupational therapy, environmental health, and pharmacy) (Geriatric Hospital Procedures, Ministry of Health, Israel). The procedures manual contains information and instructions for operating geriatric hospitals during various situations. It serves as the organizational and professional basis for activities required in various operational modes.

4 https://www.gov.il/he/Departments/DynamicCollectors/geriatric-regulations?skip=0.
